# The Chemists' Exhibition

**Published:** 1897-08-21

**Authors:** 


### The Chemists' Exhibition.

This exhibition, organised by the *British and Colonial Druggist*, was opened in the Covent Garden Theatre on Monday last. Every available space on the ground floor is occupied by stalls, and some have even overflowed into the staircase, boxes, and foyer.

Many of the exhibits were recently noticed in our columns in connection with the Medical, Surgical, and Hygienic Exhibition, notably those of Messrs. Maw, Son, and Thompson, Messrs. Bailey and Son, Vimbos (Limited), the Liverpool Lint Company, and Messrs. Idris and Co. There are, however, others that deserve mention.

The first stall that meets the eye at the entrance is that occupied by Messrs. Allen and Hanburys (Limited), who present an imposing array of their well-known jujubes and pastilles. Nurses and medical men who have not already seen it should take the opportunity of examining the excellent "Allenburys'" Feeder for Infants, a graduated curved bottle open at both ends, with a pure rubber nipple, which may be turned inside out for cleaning. The bottom end is closed by a valve stopper of pure rubber, by which air is admitted, but which does not allow leakage of milk.

Hard by is the stall occupied by Messrs. Armour and Co., said to be the largest makers of pepsin in the world. A feature of this stall which is of special interest to medical men is a display of the raw animal products (thyroid gland, &c.) from which their well-known dessicated powders are made.

At Messrs. Southall Brothers and Barclay's stall may be seen, in addition to their sanitary towels and various pharmaceutical preparations, a beautifully-made little model of their cod-liver oil factory on the coast of Norway, showing the various processes through which the oil is passed.

Messrs. A. and M. Zimmermann have a unique exhibit of new materia medica, but what is of most interest to those connected with hospitals is their Alformant, a portable little lamp for distributing definite volumes of formalin gas for the disinfection and air sterilisation of rooms. The dry formalin for this lamp is made up in the convenient form of tablets.

Among the most recent additions to Messrs. Oppenheimer's list that are shown at their stall are palatinoids of thyrcocol ½ grain and thyrcocol in powder, being the active colloid matter from selected healthy sheep's thyroid glands; bipalatinoids of compound hypophosphites with creosote, and palatinoids of green ferrous carbonate (Bland's pill). These last are specially prepared for hospitals, and supplied to them at an exceedingly moderate price.

Messrs. Reynolds and Branson exhibit their many ingenious contrivances, which have proved of such service to medical men and nurses, prominent among them being their pill box shoots and bandage shoots. Their latest addition is a new ligature reel, which can be very readily sterilised.

Messrs. Hockin, Wilson, and Co. show a large and interesting variety of pharmaceutical preparations, together with a number of perfumery and toilet articles. Nurses should not omit to notice their nurses' bags and wallets.

Disinfectants are represented by Sanitas, which may be seen in all the various forms in which it is now made up.

On the first tier is the stall of the Lattice Elastic Stocking Company, to whose goods we have before had occasion to call attention.

"Ambrosia," a new preparation of extract of malt and Devonshire cream, is the feature of Messrs. Evans, Gadd, and Co.'s stall. This is designed to take the place of cod-liver oil, having the same nutritive properties, but being far more palatable and easy to digest. Having their headquarters at Exeter, this firm is enabled to obtain cream of the richest and purest quality, for which the county of Devon is famous.

Messrs. Cook and Son exhibit their well-known medicated soaps, including their "Thomson's" Antiseptic Soap, together with many varieties for toilet purposes.

The "Medicated Auto-sprays" of the Medico-Hygienic Inventions Company (Limited) are most ingenious yet simple contrivances. They are tubes which can be hermetically sealed by a brass cap of special construction, and from which medicaments in solution can be ejected simply by the heat of the hand holding the tube.

At the stall of Messrs. Glendenning and Son, of Newcastle-on-Tyne, their beef and malt wine, which has recently been subjected to a very favourable analysis by the *Lancet*, may be seen and tasted, as also their "China Serravallo," a very palatable Austrian tonic wine.

The United British Castor Oil Company, who are, we believe, the only actual makers of the drug in the United Kingdom and the only ones that can guarantee it cold drawn, exhibit samples of their Mitchell's castor oil, and of the seeds from which it is drawn.

Messrs. Blondeau's stall, like their preparations, is most artistically got up. Here may be seen the well-known Vinolia in the innumerable useful forms in which it is now manufactured. Among the latest of these are Vinolia shaving cream, which does not need the addition of water, Vinolia cream for eczema, Vinolia violet powder, and liquid dentifrice.

Mr. John Milne makes a speciality of Lord Lister's dressings and surgical appliances, among the most noticeable being their double cyanide of zinc gauze and dressing, and ligatures in patent tubes.

Messrs. Burroughs and Wellcome's specialities are too well known to need more than a mere mention here, but visitors should note on their stall an historical tabloid medicine case which went through the Ashanti War.

The exhibition is open from one till ten p.m., and excellent music and refreshments are provided in the theatre.

